# Developing a training program of knowledge and skills in the context of administrative involuntary hospitalization: a pilot trial

**DOI:** 10.3389/fpsyt.2025.1644958

**Published:** 2025-11-10

**Authors:** Akihiro Shiina, Yusuke Sudo, Yodai Suzuki, Yu Kamata, Tomihisa Niitsu, Chiyo Fujii

**Affiliations:** 1 Division of Medical Treatment and Rehabilitation, Chiba University Center for Forensic Mental Health, Chiba-shi, Chiba, Japan; 2 Division of Clinical Neuroscience, Chiba University Center for Forensic Mental Health, Chiba-shi, Japan; 3 Department of Psychiatry, Chiba Aoba Municipal Hospital, Chiba-shi, Japan; 4 Chiba Hospital, Funabashi-shi, Japan; 5 Department of Psychiatry, Chiba University Graduate School of Medicine, Chiba-shi, Japan; 6 Department of Community Mental Health & Law, National Center of Neurology and Psychiatry, Kodaira-shi, Japan

**Keywords:** risk assessment, administrative involuntary hospitalization, mental health and welfare act, forensic psychiatry, motivation

## Abstract

**Introduction:**

To deal with offenders with mental disorder, the administrative involuntary hospitalization (AIH) scheme was adopted in Japan in 1950. However, its outcomes as well as standard of AIH adaptation remain obscure. Recently we developed a training program for improving the skills for AIH decision-making for early-career psychiatrists. The aim of this pilot study is to implement and investigate the effectiveness of this program.

**Methods:**

Through snowball sampling, this open interventional exploratory pilot study recruited licensed physicians who possessed or intended to acquire a designated psychiatrist license. Eighteen physicians were enrolled and attended a 4 h seminar including group discussion following a self-learning video session. Subsequently, they completed the Mental Health and Welfare Act Administrative Involuntary Hospitalization Test (AIH Test). An unpaired t-test was used for analysis of the AIH Test scores.

**Results:**

In total, 17 participants (mean age, 35.6 ± 5.5 years) completed the post-seminar questionnaire on the effectiveness of the seminar. The mean ± standard deviation AIH Test score improved from 12.1 ± 2.1 before the seminar to 14.7 ± 3.9 after the seminar (degrees of freedom = 34, t = −2.45, P = 0.019 [two-tailed]), indicating a significant improvement in knowledge of AIH. As a secondary outcome, scores on the Academic Motivation Scale, reflecting the motivation to study forensic psychiatry, did not change significantly before and after the seminar.

**Discussion:**

Our AIH skills training program improves knowledge of AIH, without affecting the motivation to study forensic psychiatry.

## Introduction

In many countries, the management of offenders with mental disorders has been a subject of debate among general and forensic mental health professionals. Numerous experts acknowledge that punishing such offenders is unlikely to deter recidivism, particularly in cases where the offense was committed under the direct influence of psychiatric symptoms. Instead, appropriate psychiatric treatment is necessary to prevent reoffending and facilitate reintegration into society (1, 2).

In Japan, any criminal acts committed while in a state of insanity cannot be punished, according to Article 39 of the Penal Code. However, before reaching trial, prosecutors rarely pursue cases where criminal responsibility is doubtful. Instead, in serious cases such as murder or arson, prosecutors invoke the Medical Treatment and Supervision Act (enforced in 2005) to refer the individual to a specialized court. In cases involving less serious offenses, such as theft or vandalism, prosecutors request that a public health center subject the individual to administrative involuntary hospitalization (AIH), a scheme established in 1950.

The AIH scheme was later incorporated into the Mental Health and Welfare Act, which was most recently amended in 2024, without undergoing significant revisions. Under this system, if an individual is deemed to be at risk of harming themselves or others because of a mental disorder, the prefectural governor can order their hospitalization in a designated psychiatric hospital on the basis of an evaluation by two designated psychiatrists.

However, there are few published reports on the performance and outcomes of AIH. In particular, the criteria for AIH remain vague. The Ministry of Health, Labor and Welfare issued official guidelines in 1961 titled Guidelines for Handling Administrative Involuntary Hospitalization and Consent Hospitalization for Patients with Mental Disorders. Although the “consent hospitalization” scheme was abolished in 1988, while AIH remains in place, the guidelines themselves—setting the standards for involuntary hospitalization—have remained in effect without revision or critical review.

Furthermore, there are almost no formal educational programs for designated psychiatrists to develop the necessary skills for conducting AIH evaluations. According to our recent research, most Japanese psychiatrists have received no formal training in AIH assessments. Instead they have typically acquired knowledge informally, for example through unstructured instruction from senior colleagues. Many of the participants of the research emphasized the need of structured training programs (3). Based on a questionnaire survey of designated psychiatrists, Nemoto et al. argued that the quality of professionals involved in AIH should be improved for optimized AIH management (4).

Given the current situation in Japan, we believe that a standardized training methodology for AIH evaluation and decision-making is essential for early-career psychiatrists. To our knowledge, there is no published research in Japan on educational or training methods for developing the knowledge and skills required for AIH evaluations. Accordingly, we initiated the development of a pilot training program tailored to Japan’s legal and cultural context, by adapting core principles from the international literature and aligning the design with expert opinion.

Internationally, there has been sustained debate and programmatic work on how best to educate early-career psychiatrists to acquire skills in violence risk assessment and in evaluating the need for involuntary hospitalization (5). The American Academy of Psychiatry and the Law proposed a four-year postgraduate training program for general psychiatrists, incorporating topics in forensic psychiatry each year in which mock trials are also adopted as an alternative to experiential training (6). Trained psychiatrists outperform untrained residents in predicting violence risk among psychiatric patients (7). However, the optimal ways to deliver such training within limited time constraints remain to be established. For example, McNiel et al. conducted a 5-hour workshop including pre- and posttests, didactic presentations on law and psychiatry, and small-group discussions, and observed improvements in the quality of documentation of violence risk assessments (8). They emphasized the importance of structured training programs for improving clinical psychiatric skills.

More broadly, for general psychiatrists to acquire forensic psychiatry skills, they must also be trained in the ability to objectively assess patients from a neutral standpoint. Raharjanti et al. emphasized the importance of utilizing clinical reasoning with minimized cognitive bias in determining forensic decision-making (9). Pinals et al. identified three stages with respect to the learning objectives of forensic psychiatry fellowship programs: transformation, growth of confidence, and identification and realization. They also emphasized the effectiveness of group supervision on the basis of group discussions as a method for supervising trainees (10).

Based on these principles and prior reports, we initiated the development of a training program for AIH assessments that included group discussions. As a pilot, we created a package consisting of one hour of video-based learning and four hours of in-person lectures and exercises. It is structured to facilitate an understanding of the role of an AIH evaluator during the initial lecture segment, followed by case-based discussions and group exercises designed to help participants acquire and internalize assessment skills.

The purpose of the present study is to evaluate, as a pilot study, the effectiveness of the program we developed.

## Materials and methods

### Study overview

This study is an open interventional exploratory pilot study aimed at implementing an AIH skills training program for early-career psychiatrists and evaluating its effectiveness.

### Criteria for participation

Participants were required to meet either of the following criteria:

A: Physicians who obtained their MD license at least 4 years prior to enrollment in the study and intend to obtain a designated psychiatrist license.
B: Physicians who already hold a designated psychiatrist license but have never attended a renewal seminar (5 years after acquirement).

### Recruitment of the participants

Participants were recruited using a snowball sampling method. In addition, recruitment posters were sent to psychiatric hospitals and clinics in Chiba Prefecture in Japan. Recruitment was also conducted through verbal announcements and online advertisements to maximize participation. As a pilot study, we did not perform power calculations in advance.

When a potential participant expressed interest in joining the study, the researchers provided an electronic document outlining the study details and procedures. Participants were asked to carefully review the document and provide their consent by signing and returning the consent form, including electronic submission if applicable. Informed consent was considered obtained upon receipt of the signed document. Participants retained the right to withdraw their consent at any time.

### Seminars

We held the same seminars twice: the first was held on 23 February 2023, and the second on 2 February 2025. Each seminar lasted for 4 h. Prior to attending the seminar, participants were required to complete approximately 60 min of self-study using pre-recorded lecture videos that we created in advance.

The seminar included the following components:

1. Lectures on the Mental Health and Welfare Act, covering legal provisions relevant to involuntary hospitalization.
2. Expert consensus on AIH assessments, including procedural guidelines before and after psychiatric evaluations.
3. Lectures on risk assessment for self-harm and harm to others on the basis of established criteria.
4. Case-based discussions: Participants were presented with simulated AIH assessment cases and engaged in group discussions. Each group presented their conclusions, followed by facilitator-led feedback sessions.

### Measures

A pre-training questionnaire survey was administered to all participants, followed by the training seminar on AIH assessment techniques. A post-training questionnaire survey was conducted to assess the effectiveness of the seminar by evaluating differences in responses before and after participation.

The primary outcome measure was the score on the Mental Health and Welfare Act Administrative Involuntary Hospitalization Test (hereafter referred to as the “AIH Test”). The AIH Test consisted of 20 true-or-false questions regarding the AIH scheme, designed to reflect legal provisions and common misconceptions among designated psychiatrists at the time of the training. The test items were selected and validated by three researchers on the basis of the current Mental Health and Welfare Act and related regulations.

The secondary outcome measures included scores on the Academic Motivation Scale (AMS), which evaluates intrinsic motivation, extrinsic motivation, and amotivation. The AMS consists of 28 items rated on a Likert-type scale. It categorizes motivation into intrinsic motivation (to know, to accomplish, and experiential stimulation), extrinsic motivation (identified, introjected, and external regulation), and amotivation. Each subcategory consists of four self-assessment items each rated on a 7-point scale, such that scores are in the range of 4–28 points. Prior work has supported its internal consistency and construct validity across higher-education samples, including medical students (11–13).

### Statistical analyses

We statistically analyzed the data using SPSS for Windows version 28 (IBM, Armonk, NY, USA). The significance level was set at 5%. The effect size of the intervention was estimated using Hedges’ g, which corrects for small sample bias.

### Ethics

The study protocol was approved by the Ethics Committee of Chiba University Graduate School of Medicine (Approval Nos. 10686 [28 November 2023] and M10820 [16 January 2025]). All participants provided written informed consent before enrollment. The study was registered in the UMIN Clinical Trials Registry (UMIN000050281, UMIN000056964).

## Results

A total of 18 participants completed the pre-seminar questionnaire, with 13 attending the first seminar and 5 attending the second seminar. Of these participants, 17 completed the post-seminar questionnaire, yielding a completion rate of 94%.

The mean age of the participants was 35.6 ± 5.5 years, and their mean clinical experience was 7.1 ± 1.6 years. Among the participants, nine were designated psychiatrists, whereas nine were non-designated psychiatrists.

The first seminar had 13 participants, all of whom completed both the pre- and post-seminar questionnaires. The second seminar had five participants, but only four completed the post-seminar questionnaire. For the primary analysis, we applied a conservative missing-data rule: for the one participant with missing post-seminar data, the post-seminar score was set to the minimum possible value for the instrument (e.g., 0 for the AIH Knowledge Test), which biases estimates toward the null.

The mean ± standard deviation of the AIH Test score immediately after obtaining consent was 12.1 ± 2.1, while the mean post-seminar score was 14.7 ± 3.9.

An F-test for equality of variances yielded a p-value of 0.006, indicating a violation of the assumption of equal variances. Therefore, an unpaired t-test assuming unequal variances was conducted (degrees of freedom = 34, t = −2.45, p = 0.019 [two-tailed]). This result indicates a statistically significant improvement in test scores following the seminar. The effect size was also medium to large (Hedges’ g = 0.64), based on the mean difference of 2.56 ± 3.79.

Regarding motivation scores, as shown in **Table 1** below, no statistically significant changes were observed before and after the seminar.

**Table 1:** Changes in academic motivation scale scores.

	Before	After	P-value
Intrinsic			
To know	17.6 ± 4.2 (15.6 – 19.6)	18.1 ± 5.1 (15.9 – 20.5)	N.S.
toward accomplishment	14.8 ± 4.7 (12.5 – 16.9)	16.8 ± 4.8 (14.6 – 19.0)	N.S.
to experience stimulation	13.3 ± 4.9 (11.3 – 15.6)	15.8 ± 4.6 (13.6 – 17.9)	N.S.
Total	45.7 ± 12.7 (39.9 – 51.7)	50.8 ± 13.6 (44.4 – 57.0)	N.S.
Extrinsic			
identified	20.4 ± 4.9 (18.0 – 22.7)	21.8 ± 4.1 (19.9 – 23.8)	N.S.
introjected	11.9 ± 5.1 (9.6 – 14.2)	12.9 ± 4.7 (10.7 – 15.1)	N.S.
external regulation	15 ± 6.1 (12.3 – 17.9)	16.2 ± 6.6 (12.9 – 19.4)	N.S.
Total	47.9 ± 14.0 (41.0 – 54.0)	50.9 ± 13.0 (44.5 – 56.8)	N.S.
Amotivation	8.4 ± 4.4 (6.5 – 10.4)	8.6 ± 4.5 (6.47 – 10.7)	N.S.

X ± Y denotes mean ± SD; parentheses indicate 95% CIs.

N.S., not statistically significant.

## Discussion

We developed a training package aimed at enhancing the skills required to assess the necessity of AIH and conducted an open study to evaluate its effectiveness. The results demonstrated a statistically significant improvement in participants’ knowledge regarding AIH. However, no significant changes were observed in their motivation to study forensic psychiatry.

We constructed a training package referring prior literature and programmatic reports noted above. Our findings are broadly consistent with McNeal’s report suggesting the effectiveness of 5-hour structured workshop (8), although evaluation methods differ between studies.

On the other hand, Pinal et al. proposed three-stage developmental model for forensic psychiatrists as mentioned above (10). Through our brief program, participants appeared to achieve elements consistent with the stage one - basic understanding of forensic mental health. By contrast, stage two (“growth of confidence”) is characterized by increasing clarity and comfort with role delineation, which typically requires more time and supervised experiential learning. Given that participants’ motivation scores did not increase in the present study, achieving stage-two objectives will likely necessitate a longer and dedicated curriculum.

Training in forensic psychiatry is inherently more challenging to design compared with general clinical psychiatry. One of the primary concerns is the ethical implications of using forensic patients as training participants. To address this issue, simulations using hypothetical cases have been recognized as a practical and effective educational approach (14). In this study, we implemented vignette models for AIH assessment simulations based on previous research. In recent years, training programs using virtual reality have also been explored. However, the number of such programs remains limited, and evidence regarding their educational efficacy is scarce (15).

To the best of our knowledge, this is the first study in Japan to explore methodological approaches for improving AIH assessment skills. Similar initiatives have been undertaken in other countries. For example, Laureano et al. conducted a literature review and identified a major challenge in the decision-making process for involuntary hospitalization: despite its significant impact on patients’ rights, effective communication regarding the justification for involuntary hospitalization is often lacking (16). Sugiura et al. analyzed 37 studies from 11 countries and found that although mental health professionals commonly justified involuntary hospitalization on the basis of the potential harm or threats posed by the patient, this rationale was not necessarily accepted by patients and their families (17).

Conversely, Hsiung et al. developed a training protocol to improve psychiatrists’ communication skills when informing patients about the decision for involuntary hospitalization. In that study, psychiatrists attended a lecture and then participated in a workshop where they practiced communicating involuntary hospitalization decisions to simulated patients. Post-training assessments showed that participants gained greater confidence in delivering these explanations and exhibited behavioral changes aimed at improving their communication (18). These findings suggest that structured, short-term educational sessions can influence psychiatrists’ behavior, aligning with the results of our study.

Moreover, appropriate risk assessment requires not only knowledge and technical proficiency in forensic psychiatry but also training to mitigate cognitive biases in decision-making. Sattar conducted a survey using hypothetical cases and found that psychiatrists who were more tolerant of risk-taking were less likely to opt for involuntary hospitalization. Conversely, psychiatrists who feared making errors were more inclined to choose hospitalization excessively (19). In India, Perugu et al. recently introduced competency-based medical education for medical students and evaluated its impact. Their study revealed a significant reduction in the stigma toward psychiatry among students (20). These findings suggest that structured training programs can reduce stigma among healthcare providers, which in turn may not only enhance medical ethics but also improve the accuracy of decision-making regarding involuntary hospitalization.

In this study, we adopted a training format in which participants watched simulated AIH assessment cases, engaged in group discussions, and received facilitator feedback. In other countries, more realistic approaches have been implemented, such as simulated assessments using standardized patients, who also provide scoring and feedback. Although such advanced methods are expected to enhance learning outcomes, they are associated with higher costs, and there is limited evidence regarding their cost–benefit balance.

The participants in this study underwent a 1 h pre-training video session followed by a 4 h seminar, resulting in a significant improvement in their AIH assessment knowledge. This result suggests that a well-structured training package can effectively enhance risk assessment skills within a short period.

However, no significant changes were observed in participants’ motivation. Motivation is generally classified into intrinsic and extrinsic motivation, with intrinsic motivation being less susceptible to external influence. In a previous study also using the AMS, we similarly found insufficient evidence supporting an increase in motivation. There is currently no psychometrically validated threshold for interpreting AMS scores. According to a comparative study of university students in Japan and New Zealand, students in Japan scored higher than the present samples on the Intrinsic Motivation “to Know” subscale (21.81 ± 4.27), whereas their scores on “toward Accomplishment” (11.38 ± 4.22) and “to Experience Stimulation” (11.83 ± 4.16) were lower than those observed in this study (21). Conversely, in a study of Hungarian medical students marked far higher motivation than our samples (22). Taken together, we cannot conclude that the seminar we implemented increased participants’ intrinsic motivation toward forensic psychiatry.

This study has several limitations. First, as a small pilot sample, the study is underpowered to detect small-to-moderate effects, and the generalizability of the findings is also limited.

Second, the primary outcome measure of the present study was a true-or-false knowledge test score, which may not be enough for objectively evaluating AIH assessment skills. Accordingly, improvements in actual assessment performance cannot be inferred from these data. Ideally, standardized patient assessments with structured scoring performed by examiners would be preferable. Because there is no standardized AIH assessment protocol or scoring criteria in Japan, we had to adopt an objective paper-based test as an alternative. In future work, we plan to incorporate standardized performance-based assessments with blinded raters to evaluate AIH assessment quality more directly.

Third, as an open study, this research likely attracted participants with high motivation for learning AIH assessment skills. Also, as we gathered the participants with snowball sampling, it was possible that some enthusiastic groups for learning forensic psychiatry were constructed in the seminar. Consequently, the effectiveness of the training may have been overestimated. This is a major limitation of this study due to the study design. At the same time, the participants may have had a higher baseline level of knowledge, which could also explain the lack of significant changes in motivation because of the ceiling effect. For accurate validation of the training’s effectiveness, a large-scale, randomized controlled trial with a less biased sample is necessary.

In conclusion, we developed a training package aimed at improving AIH assessment skills in Japan and conducted an open trial to evaluate its learning effectiveness. The results showed a significant improvement in participants’ knowledge of AIH assessment; however, no significant changes in motivation were observed. These findings suggest that even a short but structured training program can enhance early-career psychiatrists’ risk assessment skills and understanding of the Mental Health and Welfare Act. Because this study is a pilot open trial, to further validate the effectiveness of this program, a larger-scale randomized controlled trial is necessary.

## Data Availability

The raw data supporting the conclusions of this article will be made available by the authors, without undue reservation.
